# Self-reported sleep quality and sleep disorders in people with physician-diagnosed gout: an Internet cross-sectional survey

**DOI:** 10.1186/s13075-019-1821-2

**Published:** 2019-01-25

**Authors:** Jasvinder A. Singh

**Affiliations:** 1 Medicine Service, Birmingham VA Medical Center, Birmingham, AL UK; 2 Department of Epidemiology at the School of Public Health, University of Alabama at Birmingham, Birmingham, AL 35294 USA; 3 Department of Medicine at the School of Medicine, University of Alabama at Birmingham, 510 20th Street S, Faculty Office Tower 805B, Birmingham, 35294 USA

**Keywords:** Gout, Internet, Survey, Sleep, Sleep disorders, Sleep apnea, Quality improvement

## Abstract

**Background:**

Limited information exists regarding sleep disorders in gout. Our objective was to assess the burden of sleep disorders in people with gout.

**Methods:**

A brief anonymized Internet survey of people with physician-diagnosed gout who visited a gout education website assessed the frequency of sleep problems, sleep quality over the past 24 h (0 = best possible sleep, 10 = worst possible sleep), daytime sleepiness on a typical day (0 = none and 10 = most sleepy during the day), sleep quantity (number of hours of sleep), and the frequency of snoring or gasping, and snorting or stopping breathing during the sleep, using validated questionnaires, including the NHANES 2016 sleep questionnaire. We used Chi-square test to compare the categorical and *t* test the continuous variables.

**Results:**

Of the 454 website visitors who clicked the survey, 320 survey respondents reported physician-diagnosed gout. Mean age was 57 years (standard deviation [SD], 13.4), 72% were male, 77% were White, and mean gout duration was 7.6 (SD, 11). Of the respondents, 23% reported doctor-diagnosed sleep disorder (sleep apnea, 17%; sleep study ordered, diagnosis pending, 4%; other sleep disorder 2%). A mean 6.7 h of sleep per night was reported (SD, 1.3). Eighty-six percent reported snoring during sleep and 45% reported having snorted, gasped, or stopped breathing while asleep. Two thirds of the patients reported feeling sleepy during the day, at least 3–4 times a month or more. Sleep quality was 5.5 (SD, 2.6), and daytime sleepiness was 3.5 (SD, 2.6) on a 0–10 scale (higher = worse).

**Conclusions:**

People with physician-diagnosed gout reported frequent sleep disorders and daytime sleepiness in an Internet survey. More in-depth studies are needed to better understand the association of gout with sleep disorders.

## Background

Sleep disorders constitute a major public health burden for the US adults with a prevalence of 6% according to the National Health and Nutrition Examination Survey (NHANES) 2005–2006 [1], obstructive sleep apnea (OSA) being the most common type [2]. Undiagnosed and un- or under-treated OSA was estimated to cost the U.S. $150 billion in 2015 according to a recent report [3], and the health care savings with proper diagnosis could be $100 billion [4]. OSA is associated with significant morbidity [5, 6] and reduced work productivity [7]; cardiovascular morbidity may be related to oxidative stress and inflammation [8]. Effective treatments are available for OSA and other sleep disorders that can reduce morbidity and improve quality of life and work productivity [9–11].

Both gout [12] and sleep apnea [13] (a common form of sleep disorder) are associated with metabolic syndrome, defined as the presence of glucose intolerance, hypertension, atherogenic dyslipidemia, and obesity [14]. A recent case-control matched the UK study that used primary care/general practice data reported that gout was associated with 1.4-fold higher risk of any sleep problem, with 50% higher risk of OSA and 37% higher risk of non-sleep apnea problems [15]. An analysis of the 5% random US Medicare data showed that gout was independently associated with a 2-fold higher risk of OSA, after adjusting for demographics, comorbidity, cardiovascular disease, and medications used for the treatment of gout and cardiovascular disease [16]. Another study examined the Health Improvement Network (THIN) database in the UK and found that the incidence of gout was increased by 50% in people with vs. without baseline OSA [17], indicating the OSA may be a risk factor for gout. A study of the US Medicare population, mostly 65 years or older, revealed that gout was associated with a 2-fold higher risk of incident OSA [18]. These study findings indicate that gout and OSA are associated with each other, likely related to both sharing metabolic syndrome comorbidities.

Several biologic mechanisms potentially link OSA to a higher risk of gout and gout flares, including hypoxia associated with OSA leading to enhanced nucleotide turnover subsequently leading to hyperuricemia, and increased urinary urate to creatinine ratio [19–21] or possible activation of pathways that accelerate T cell immune response can increase the risk of development of cell-mediated autoimmune disease such as gout [22]. Conversely, gout-associated oxidative stress and acute and chronic inflammation through NALP3 (NACHT, LRR and PYD domains-containing protein 3) inflammasome activation and cytokine activation can put an individual at risk for developing OSA, since these pathways are activated in both these conditions [23–27]. The exact mechanisms for association either way have not been defined and will need careful examination with basic, clinical and translational research. A long latent period for both conditions, gout and OSA, makes the determination of cause-and-effect difficult, if not impossible.

Gout is the most common inflammatory arthritis that affects 4% Americans, is often symptomatic and 63% of gout patients have metabolic syndrome comorbidities [12, 28], risk factors for OSA. A great majority of patients affected with OSA remain undiagnosed [29, 30], a major quality gap [2]. Gout, if shown to be associated with OSA, can serve an early indicator of sleep apnea and help its diagnosis [31, 32]. Recent qualitative studies in patients with gout identified that sleep disorders are experienced commonly and likely contribute to quality of life (QOL) deficits in these patients [33, 34]. If sleep disorders were found to be common in people with gout, a quality improvement (QI) initiative in the gout clinic would be to screen gout patients for sleep disorders and thus improve the clinic intake process for effective screening of an important comorbidity. Early diagnosis and appropriate management of sleep disorders (including OSA) in people with gout can fill two quality gaps (diagnosis, management) and may improve outcomes in people with gout and gout/OSA overlap [9, 10]. Due to limited information available for sleep disorders in gout, this quality improvement (QI) initiative was conducted to examine the frequency of sleep problems and disorders in people with gout, using an anonymized Internet survey.

## Methods

People visiting the Gout and Uric Acid Education Society’s (GUAES) website (http://gouteducation.org) were invited to participate in a brief anonymized Internet survey from January to October 2017. People who reported physician-diagnosed gout were invited to respond to a survey that queried people about demographics (age, gender, race/ethnicity using the NIH Collaboratory definition [35]), the duration of gout diagnosis, gout flares, the number of medical comorbidities (including gout), and overall symptoms from gout and receipt of and adherence (the number of days medication was missed) to prescribed urate-lowering therapy (ULT; allopurinol or febuxostat). Questions regarding gout duration and gout flares were the same as used previously by us [36]. The number of gout flares’ question was similar to that used previously for patient-reported flare as part of validated gout flare definition [37], in addition providing some details of a gout flare (“How many gout flares did you have in the last year? A typical gout flare is associated with severe pain, swelling and inability/difficulty to move the joint, and lasts 7–10 days without treatment”).

The frequency of sleep problems including sleep apnea was assessed. Sleep quality was assessed using a single-item validated patient-reported Sleep Quality Scale [38] that assessed the quality of sleep over the past 24 h on a 0 (best possible sleep) to 10 (worst possible sleep) scale. Daytime sleepiness was assessed with a single-item validated question, asking patient about daytime sleepiness on a typical day with 0 meaning none and 10 being the highest, i.e., the most sleepy during the day [39]. Other sleep quality and quantity outcomes were assessed using the NHANES 2016 sleep questionnaire that included the number of hours of sleep, and the frequency of snoring; gasping, snorting, or stopping breathing during the sleep; and feeling excessively/overly sleep during the day [40].

As a QA/QI initiative, this anonymized Internet survey did not require an approval from the Institutional Review Board. No written informed consent was needed since the anonymized survey was offered for voluntary participation at the GUAES patient education website informing people that they could click out of the survey if not interested, this will not directly benefit them and they can stop participating at any time. Chi-square test were used for categorical and t-test for continuous variables. A *p*-value < 0.05 was considered statistically significant.

## Results

Of the 454 website visitors who clicked the survey, 320 survey respondents reported physician-diagnosed gout. The mean respondent age was 57 years (SD, 13.4), 72% (166/232) were male, and 77% (179/232) were White (Table 1). Gout duration, gout flares in the last month, and the number of medical comorbidities (including gout) were 7.6 years (SD, 11), 1.5 flares (SD, 1.3), and 2.7 (2.6), respectively (Table 1). Only 16% (45/286) reported no gout flare in the last year, and only 10% (24/241) reported mild or no symptoms of gout.

**Table 1:** Characteristics and disease severity of study cohort of people with gout (*n* = 320)

	*N* (%), unless specified otherwise
Demographics	
Age, mean (SD)	57.0 (13.4)
Male sex	166 (72%)
Race/ethnicity^1^	
White	179 (77%)
Black or African American	13 (6%)
Asian	24 (10%)
American Indian or Alaskan Native	1 (0.4%)
Native Hawaiian or other Pacific Islander	13 (6%)
Other	13 (6%)
Hispanic ethnicity^1^	24 (10%)
Gout: disease, flare*, treatment, and comorbidity characteristics	
Gout duration, years, mean (SD)^2^	7.6 (10.8)
Gout flares in the last month, mean (SD)^3^	1.5 (1.3)
Gout flares in the last year, mean (SD)^3^	5.2 (6.1)
Gout flares in the last month^3^	
None	58 (20%)
1	115 (40%)
2	69 (24%)
3	20 (6%)
4	11 (3%)
5	17 (5%)
Gout flares in the last year^3^	
None	45 (16%)
1	51 (18%)
2	36 (13%)
3	37 (12%)
4	13 (4%)
5	19 (6%)
≥ 6	85 (30%)
Doctor prescribed allopurinol or febuxostat^3^	155 (54%)
Days in the last month missed a dose of allopurinol or febuxostat, mean (SD)^4^	8.0 (12.3)
Days in the last month missed a dose of allopurinol or febuxostat^4^	
0	72 (51%)
1	6 (4%)
2	8 (6%)
3	9 (6%)
4	3 (2%)
5	4 (2%)
≥ 6	39 (28%)
Number of medical conditions diagnosed, including gout, mean (SD)^5^	2.8 (2.6)
Number of medical conditions diagnosed, including gout ^5^	
1	97 (38%)
2	50 (20%)
3	52 (20%)
4	21 (8%)
≥ 5	35 (14%)
How bad are the symptoms from your gout overall?^6^	
Very severe (as bad as it can be)	58 (24%)
Severe	102 (42%)
Moderate	57 (24%)
Mild	17 (7%)
No symptoms from my gout	7 (3%)

ULTs include prescription of one of the following: allopurinol (also called Aloprim or Zyloprim) or febuxostat (also called Uloric) or probenecid (benemid). Missing data for each question from the sample of 320 respondents: ^1^*n* = 88; ^2^*n* = 21; ^3^*n* = 30, 34, 35, respectively, for these three questions ^4^*n* = 179, ^5^*n* = 65, and ^6^*n* = 79

*Gout flare was defined using a question similar to that used previously for patient-reported flare and in addition providing some details of a gout flare: “How many gout flares did you have in the last year? A typical gout flare is associated with severe pain, swelling, and inability/difficulty to move the joint and lasts 7–10 days without treatment”

Of the respondents, 23% (56/241) reported doctor-diagnosed sleep disorder, which was sleep apnea (17%) or possible sleep apnea (4%, sleep study ordered, diagnosis pending) in the majority and other sleep problems in 2% (Table 2). Sleep quality on a 0–10 single-item scale (10 = best possible sleep) over the past 24 h was 5.5 (SD, 2.6). Daytime sleepiness on a 0–10 single-item scale (10 = most daytime sleepiness) was 3.5 (SD, 2.6).

**Table 2:** Self-reported sleep characteristics of people with physician-diagnosed gout

	All, *N* (%)*
Doctor or health care provider diagnosed the following sleep disorder	
Doctor-diagnosed sleep apnea	42 (17%)
Doctor-diagnosed sleep disorder other than sleep apnea	5 (2%)
Doctor has ordered a sleep study, no diagnosis as yet	9 (4%)
No diagnosis of sleep disorder	185 (77%)
Single-item sleep quality, mean (SD) (0 = worst possible sleep; 10 = best possible sleep)	
Over the past 24 h	5.5 (2.6)
Over the past 7 days (adapted for longer duration)	5.8 (2.3)
Single-item subjective sleepiness (SS) scale: sleepiness on a typical day, mean (SD) (0 = not sleepy at all during the day; 10 = the most sleepy during the day)	3.5 (2.6)
NHANES 2016 sleep questionnaire	
Average number of hours of sleep per night, mean (SD)	6.7 (1.3)
Snored while asleep in the past 12 months	
Never	26 (14%)
Rarely, 1–2 nights a week	45 (25%)
Occasionally, 3–4 nights a week	48 (27%)
Frequently, 5 or more nights a week	60 (34%)
Snorted, gasped, or stopped breathing while asleep in the past 12 months	
Never	86 (55%)
Rarely, 1–2 nights a week	38 (24%)
Occasionally, 3–4 nights a week	21 (13%)
Frequently, 5 or more nights a week	12 (8%)
Felt excessively or overly sleepy during the day in the past month	
Never	16 (8%)
Rarely, 1–time a month	53 (28%)
Sometimes, 2–4 times a month	76 (40%)
Often, 5–15 times a month	45 (24%)
Always, 16–30 times a month	0 (0%)

*Missing, *n* = 79–85 on most questions, except rating of the quality of sleep (*n* = 106), excessive daytime sleeping (*n* = 130) and reporting of snoring (*n* = 141), snorting/gasping/stopping breathing (*n* = 163); ^1^*p* value = 0.001; ^2^*p* value = 0.07; ^3^*p* value = 0.002

People with gout reported a mean 6.7 h of sleep per night (SD, 1.3). Only 14% (26/179) respondents never snored during the night, i.e., 86% (153/179) reported snoring during sleep with 34% (60/179) reported snoring 5 or more nights in the last week; 45% (71/157) reported having snorted, gasped, or stopped breathing while asleep (Table 2). Two-thirds of the patients reported feeling sleepy during the day, at least 3–4 times a month or more.

No age, sex, or race differences were seen in patient-reported sleep parameters, except the following: (1) compared to males with gout, females with gout reported better sleep quality in the last 7 days, 6 vs. 4.9 (*p* = 0.002), but more daytime sleepiness, 3.1 vs. 4.6 (*p* = 0.001); (2) compared to people ≥ 65 years with gout, younger people with gout reported more daytime sleepiness (*p* = 0.03) and more frequent snorting, gasping, or stopping breathing while asleep in the past 12 months (*p* = 0.01) (Appendix).

## Discussion

In an anonymized cross-sectional Internet gout survey of people visiting a gout education website, a quarter of respondents with gout reported sleep problems, with sleep apnea being the most commonly diagnosed sleep disorder. Nine out of 10 respondents reported snoring during sleep, and 5 out of 10 reported snorting, gasping, or stopping breathing while asleep. This high prevalence of snoring, snorting, gasping, or stopping breathing while asleep indicates that the actual prevalence of OSA may be even higher than the 17% physician-confirmed OSA diagnosis reported by people with gout. Even the 17% prevalence of sleep apnea in gout is much higher than the 4–7% reported in the US general population previously [2]. Our recent US Medicare study reported a 3-fold higher unadjusted and 2-fold higher adjusted hazard ratio of a new diagnosis of OSA in Medicare-recipient adults with gout, who were mostly 65 years or older [18]. The current study supports the finding of significantly higher prevalence of sleep apnea in people with gout, in an independent sample of subjects from across the world, with no geographic or age limits.

Sleep quality in gout on the single-item was much lower than that reported from two trials of pregabalin in fibromyalgia, a condition where sleep abnormalities are very common and often presenting features of the disorder, 5.5 vs. 6.2–6.7 [38]. Daytime sleepiness in this gout cohort was much lower compared to a cohort of people with diagnosed/known sleep disorders, 3.5 vs. 6.5 [39], which is not unexpected, since people with known sleep disorders would be expected to be much worse than those with a musculoskeletal disorder (gout). Frequent daytime sleepiness was reported by two thirds of people with gout, which is quite concerning. An interesting finding was that women and younger people with gout were more likely to report daytime sleepiness than their counterparts. These findings have potential clinical implications. Although these findings are not generalizable to gout cohorts reported from gout clinics, the question is whether screening gout patients for sleep disorders in the clinic can aid early diagnosis and treatment of sleep apnea and other sleep disorders and improve patient outcomes. These are important clinical questions that need further study.

So, what are the implications of these study findings for clinical practice? Knowing that both gout and OSA are common in the general population, an earlier recognition of undiagnosed OSA and sleep disorders in people with gout can improve the ability to diagnose and treat sleep disorders. Our study indicates that a clinic QI initiative of querying patients with gout about sleep disorders may be appropriate, low-cost, and practical way to improve the recognition of coexisting sleep disorders in patients with gout. Even in the absence of a subsequent sleep disorder diagnosis, recognizing the impact of gout and gout flares on sleep can lead to a patient-centered care approach. Appropriate referral of patients with severe symptoms and supporting examination findings to a sleep study for confirming/ruling out OSA and/or a sleep expert for diagnosis of OSA and other sleep disorders can fill two quality gaps of delayed/missed diagnosis and delayed treatment of OSA and other sleep disorders.

This study should be interpreted considering study limitations. Findings may only be generalizable to patients with gout who use the Internet, and not all gout patients. The Internet study design may have selected for a younger people, who might be more technologically oriented. However, the use of Internet is growing in all segments of the society, and 70% and 27% of our survey respondents were ≥ 50 and ≥ 65 years old, respectively. Response bias is an important study limitation, and missingness of responses was higher for certain variables/questions such as ULT use (missing, *n* = 179) and some sleep questions such as snorting/gasping/stopping breathing (*n* = 163) compared to that noted for race/ethnicity/sex (missing, *n* = 88). Self-reported sleep parameters are not without limitations, since over-reporting of sleep duration by self-report has been described, and self-report had only moderate correlation with objective assessment of sleep duration [41]. The lack of collection of body weight and body mass index data is another study limitation. A strength of this study was the use of validated sleep measures, including the NHANES 2016 sleep questionnaire, and limiting these analyses to only those with self-reported physician-confirmed gout.

## Conclusions

In conclusion, Internet users with self-reported physician-diagnosed gout commonly reported physician-diagnosed sleep disorders. A large proportion of patients with gout also reported frequent snoring, snorting, gasping, or stopping breathing while asleep, indicating an additional cohort with subclinical or undiagnosed OSA. Given high prevalence of sleep disorders in people with gout, further studies are needed to examine the true rates and types of sleep disorders in gout. An early diagnosis and treatment of sleep disorders in gout may reduce its impact and morbidity and may improve quality of life.

## Appendix

**Table 3:** Sleep parameters with significant differences by sex, age, or race

	Sleep quality in the last 24 h (0–10 scale, 10 = worst possible sleep), mean (SD)	Sleep quality in the last days (0–10 scale, 10 = worst possible sleep), mean (SD)	Daytime sleepiness (0–10 scale, 10 = most daytime sleepiness), mean (SD)	Average number of hours of sleep per night, mean (SD)
Sex				
Male	5.7 (2.5)	*6.0 (2.2)*	*3.1 (2.3)*	6.7 (1.2)
Female	5.0 (2.5)	*4.9 (2.3)*	*4.6 (3.0)*	6.5 (1.6)
*p* value	0.07	*0.002*	*0.001*	0.38
Age				
≤ 50 years	5.3 (2.7)	5.8 (2.4)	*4.1 (2.6)*	6.5 (1.4)
> 50 and < 65 years	5.5 (2.5)	5.7 (2.3)	*3.4 (2.5)*	6.6 (1.2)
≥ 65 years	5.8 (2.6)	5.9 (2.1)	*3.0 (2.6)*	6.9 (1.4)
*p* value	0.57	0.92	*0.03*	0.17
Race				
White	5.4 (2.5)	5.6 (2.3)	3.4 (2.5)	6.7 (1.3)
Non-White	5.8 (2.8)	6.1 (2.2)	3.9 (2.9)	6.5 (1.5)
*p* value	0.39	0.19	0.27	0.31

Italic emphasis represents statistically significant differences with a *p* value < 0.05. No significant differences were noted in doctor-diagnosed sleep disorder or in snoring during sleeping, by sex, age, or race

## Abbreviations

SD: Standard deviation
OSA: Obstructive sleep apnea
NHANES: National Health and Nutrition Examination Survey
QOL: Quality of life
QI: Quality improvement
GUAES: Gout and Uric Acid Education Society
ULT: Urate-lowering therapy
